# Pyroptosis: a new insight into intestinal inflammation and cancer

**DOI:** 10.3389/fimmu.2024.1364911

**Published:** 2024-02-22

**Authors:** Limin Chao, Wenjing Zhang, Yuchao Feng, Pei Gao, Jinyou Ma

**Affiliations:** College of Animal Science and Veterinary Medicine, Henan Institute of Science and Technology, Xinxiang, China

**Keywords:** pyroptosis, inflammasomes, intestinal inflammation, cancer, gut microbiota, intestinal mucosal barrier

## Abstract

Pyroptosis is an innate immune response triggered by the activation of inflammasomes by various influencing factors, characterized by cell destruction. It impacts the immune system and cancer immunotherapy. In recent years, the roles of pyroptosis and inflammasomes in intestinal inflammation and cancer have been continuously confirmed. This article reviews the latest progress in pyroptosis mechanisms, new discoveries of inflammasomes, mutual regulation between inflammasomes, and their applications in intestinal diseases. Additionally, potential synergistic treatment mechanisms of intestinal diseases with pyroptosis are summarized, and challenges and future directions are discussed, providing new ideas for pyroptosis therapy.

## Graphical Abstract

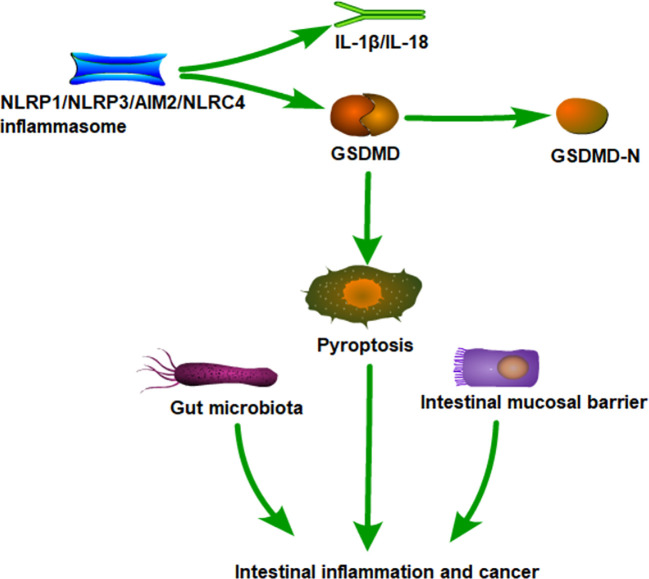

## 1 Introduction

Innate immunity serves as the first line of defense for the human body against foreign invaders. It comprises a collection of pattern recognition receptors (PRRs) encoded by the host that act as barriers against pathogen-associated molecular patterns (PAMPs) and damage-associated molecular patterns (DAMPs) (1). Dysfunction of these PRRs can contribute to infectious colitis, inflammatory bowel disease (IBD), and cancer (2). After detection of PAMPs or DAMPs by innate immunity, associated nucleotide binding oligomerization domain like receptors (NLRs) or absent in melanoma 2 (AIM2)-like receptors oligomerize into multiprotein complexes containing caspase-1. This regulates the activation of inflammatory caspases, promoting expression, maturation, and release of proinflammatory cytokines, thereby triggering an inflammatory response (3, 4). Interestingly, activated inflammatory caspases also induce an inflammatory form of regulated cell death called “pyroptosis”.

Pyroptosis is a programmed cell death triggered by inflammasomes that detect intracellular solute contamination. Scientists first discovered pyroptosis in Shigella flexneri infected macrophages (5). Currently, Salmonella, Shigella flexneri, Listeria, Pseudomonas aeruginosa, Legionella pneumophila, and Yersinia induce caspase-1 mediated macrophage death, in both monocytes and other cells (6–9). Moreover, stimulation is not limited to conserved PAMPs, some non-biological stimuli such as DAMPs, can also activate host pattern recognition receptors to trigger pyroptosis (10).

In the classical pathway, assembled NLRC4, AIM2, NLRP3, and Pyrin proteins are activated to cleave procaspase-1, generating active caspase-1. Then, GSDMD is cleaved to form a GSDMD-N-terminal fragment and translocated to the cell membrane, disrupting and causing perforation, leading to cell rupture and release of cell contents, resulting in pyroptosis. Meanwhile, active caspase-1 processes the inflammatory factor IL-1β and IL-18 into a mature form, releasing them into the extracellular matrix through the ruptured membrane, endowing pyroptosis with pro-inflammatory properties. Mature IL-1β acts as a potent pro-inflammatory mediator, recruiting innate immune cells to infection sites and regulating adaptive immune cells. Mature IL-18 promotes interferon secretion and enhances the cytolytic activity of natural killer cells and T cells, contributing to eliminating pathogenic microbial infections or abnormal cells *in vivo (* 11). Indeed, increasing evidence suggests abnormal pyroptosis is closely related to intestinal inflammation, autoimmune diseases, colorectal cancer (CRC), infectious diseases, cardiovascular diseases, and neurological related diseases (12–15). This review mainly summarizes the latest insights on the roles of pyroptosis and inflammasomes in intestinal inflammation and cancer, pointing out the direction for future research.

## 2 The classification of inflammasomes

Inflammasomes are multiprotein complexes that primarily reside in the cytoplasm of innate immune cells (16). However, they are also present in lymphocytes and epithelial cells. So far, their function has been linked to the pathogenesis of various diseases, including inflammatory diseases, metabolic disorders, neurodegenerative disorders, and cancer (17). In addition, an increasing number of studies have shown that they are involved in the development of intestinal inflammation and cancer.

The inflammasome is defined as a complex formed by PRRs, apoptosis speck-like proteins (ASC), and procaspase-1 proteins upon cellular stimulation with danger signals. It functions to cleave procaspase-1 into active caspase-1. In most cases, this is a key process in pyroptosis. Based on homology of protein domains, most PRRs are divided into five families: NOD-like receptors (NLRs), C-type (carbohydrate binding lectin domains) lectin receptors (CLRs), retinoic acid inducible gene (RIG)-I like receptors (RLRs), Toll like receptors (TLRs), and AIM2-like receptors (ALRs) (18). By different protein localization, PRRs are divided into unbound intracellular receptors and membrane bound receptors. The former includes RLRs, NLRs, and ALRs, recognizing intracellular pathogens in the cytoplasm. CLRs and TLRs are on endocytic compartments or cell membranes, recognizing microbial ligands in endosomes or extracellular spaces (19). Many proteins, such as NLRP1, NLRP3, NLRC4, and AIM2, initiate inflammasome formation, gradually attracting much attention (20).

### 2.1 NLRP1

NLRP1 was the first protein discovered as an inflammasome, primarily expressed by epithelial and hematopoietic cells (21). The NLRP1 inflammasome is a multiprotein complex comprising the NLRP1 receptor protein, the adapter protein ASC, and the effector protein caspase-1 (22). This inflammasome can activate the pro-inflammatory protease caspase-1, releasing IL-1β and IL-18 and cleaving the GSDMD protein to form pores on the cell membrane, leading to pyroptosis (23).

Many studies have investigated the role of NLRP1 in IBD pathogenesis. The report shows that compared to wild-type mice, DSS-induced colitis is more severe in Nlrp1b-deficient mice (24). However, another report’s results indicate that NLRP1 lacks the ability to inhibit DSS-induced colitis by promoting beneficial, butyrate producing Clostridium expansion (25). NLRP1 activation can exacerbate DSS-induced colitis, relating to enhanced Th1 response and increased IL-18/IFNγ production in the intestine. The latest research shows that secoisolariciresinol diglucoside alleviates colitis by inhibiting NLRP1 inflammasomes, correlating with reduced IL-1β, IL-18, and TNF-α levels in DSS-induced colitis mice (26). Therefore, NLRP1 may be a potential therapeutic target for IBD, and its contribution to colitis may relate to gut microbiota composition.

### 2.2 NLRP3

Of all NOD-like receptor inflammasomes, NLRP3 has garnered the most attention. Unlike other receptor proteins, NLRP3 is highly sensitive to various non-pathogenic factors both environmental and endogenous which can trigger abnormal NLRP3 inflammasome activation, implicated in complex pathologies like IBD, neurodegenerative diseases, and cancer (27, 28). NLRP3 comprises three structural domains: an N-terminal PYD for homotypic binding; a nucleotide binding and oligomerization domain (NACHT) and LRR domain, which indirectly recruit and activate caspase-1 with adaptor protein ASC (29). NLRP3 inflammasome activation usually requires two signals: an initiation signal and an activation signal. The initiation signal is typically provided by microbial components like lipopolysaccharide (LPS), recognized by Toll-like receptor TLR4. Alternatively, it can be endogenous molecules like IL-1β or TNF-α. Activation signals are triggered by extracellular ATP, bacterial toxins, viral RNA, etc (30). These activating factors induce cellular stress responses and activate NLRP3 inflammasomes, though NLRP3 indirectly perceives them rather than direct recognition.

Recent research suggests that NLRP3 inflammasomes are involved in regulating colonic immune homeostasis. Reports indicate that NLRP3 and NLRP6 inflammasome-mediated IL-18 production in the colon promotes downregulation of IL-22 binding proteins, blocking IL-22’s role in regulating colonic epithelial cell repair (31). Studies also find that compared to wild-type mice, mice with NLRP3 inflammasome deficiency exhibit exacerbated DSS-induced colitis and decreased epithelial integrity (32). Synthetic drugs, phytochemicals, and plant extracts exert colon-protective effects and inhibit NLRP3 and caspase-1 activation, demonstrating their role in UC inflammation (33). However, further understanding is needed on how inhibiting NLRP3 inflammasome activation can significantly treat IBD.

### 2.3 NLRC4

NLRC4 belongs to the NLR family and is composed of an N-terminal CARD, NACHT domains, and C-terminal LRR domains. It is activated by bacterial flagella proteins and flagella related secretory system components. NLRC4 activation requires the neuronal apoptosis inhibitory protein (NAIP) to sense bacterial flagellin or the type 3 secretion system (34). It has been determined that NLRC4 is crucial for pathogen restriction in phagocytic cells of the intestinal lamina propria and other sites (35). NLRC4 deficient mice induce more tumors in the AOM/DSS model, but are not related to inflammation and colitis severity in mice is similar to wild-type mice. Conversely, NLRC4 deficiency increases epithelial proliferation in advanced tumors and reduces apoptosis (36). The latest study found that flagellin coupled proteins regulate inflammatory cytokine secretion by macrophages via NLRC4 and NLRP3 inflammasome activation (37). However, the roles of NLRC4 in intestinal inflammation and cancer remain ill-defined.

### 2.4 AIM2

AIM2 is a cytoplasmic dsDNA sensor and an ALR inflammasome sensor (38). Under normal conditions, AIM2 is autoinhibited. Cytoplasmic DNA from damaged host tissues, viruses, or microorganisms binds AIM2’s HIN-200 domain, exposing AIM2’s PYD and recruiting adaptor ASC, activating caspase-1 (39). In IBD patients’ small and large intestines, AIM2 mainly expresses in epithelial cells and macrophages, indicating potential roles in intestinal inflammation. In addition, studies have found that AIM2 protects mice from DSS induced colitis, while Aim2 deficient mice are more susceptible to colitis. AIM2 activates inflammasomes by sensing bacterial DNA in the gut, playing a crucial role in regulating gut microbiota and inflammation (40). The AIM2 gene contains a microsatellite instability site, potentially mutating in CRC and small intestine cancer (41). A recent study found AIM2 inhibits BRAF mutated CRC growth via caspase-1 *in vitro* and *in vivo*, confirming AIM2 overexpression significantly inhibits BRAF-mutated CRC tumor growth and metastasis. Further research found that AIM2 inhibited BRAF mutated CRC cell growth via caspase-1 activation (42). Pyroposis may be involved through AIM2 inflammasomes, but further research is needed to verify this.

### 2.5 Inter-regulation of inflammasomes

The diversity of inflammasome structure and function can be supplemented by the regulation of other inflammasome components. Many inflammasome components interact to activate or inhibit other inflammasomes, thereby regulating their function.

**Figure 1:**
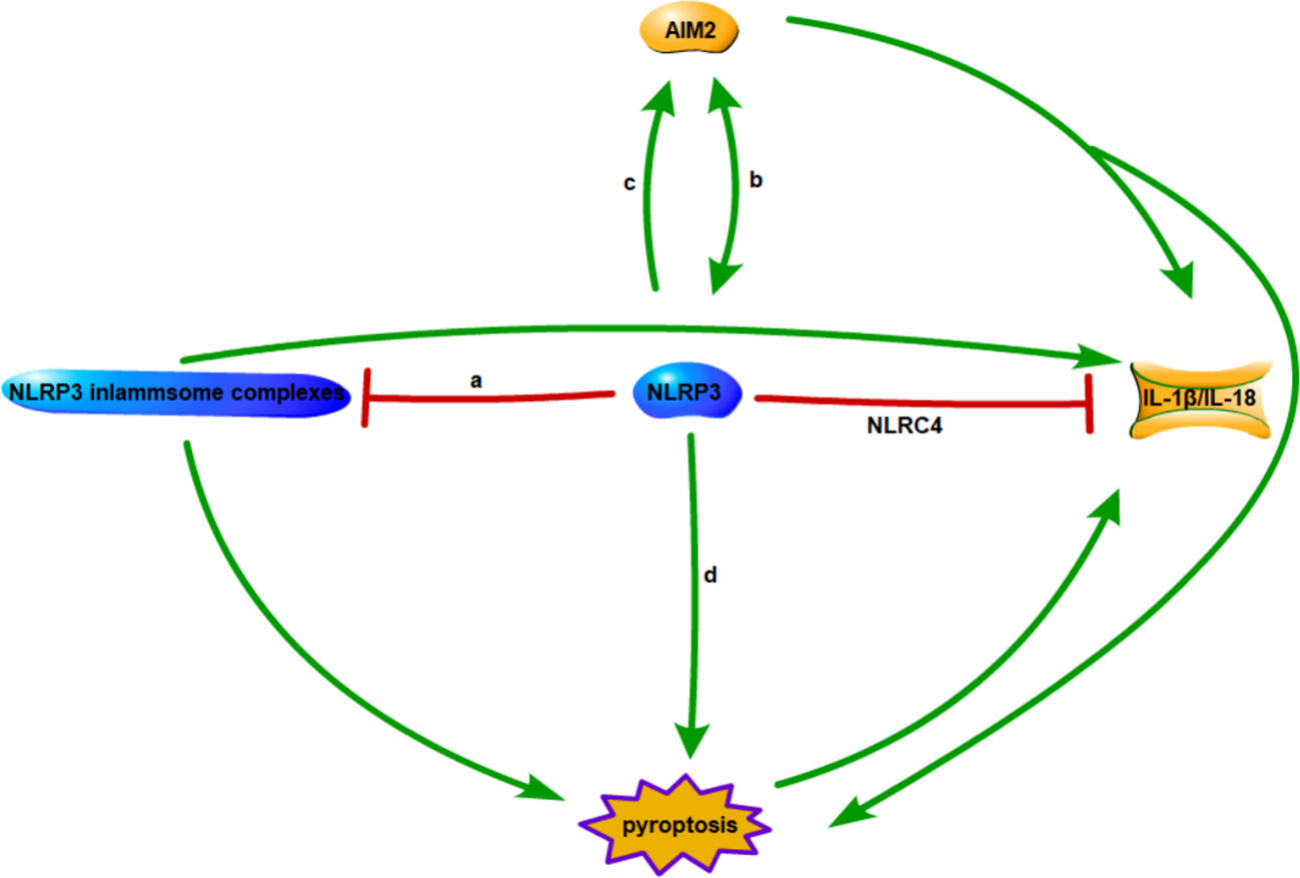
Interactions between inflammasomes. **(A)** Competitive combination of NLRC3 and NLRP3 with ASC. **(B)** AIM2 inflammasome is a compensatory pathway for NLRP3 inflammasome mediated macrophage pyroptosis. **(C)** AIM2 amplifies infection signals to trigger activation of NLRP3 inflammasomes. **(D)** NLRP12/NLRP3/NLRC4 synergistically triggers pyroptosis.

Multiple studies have demonstrated the inhibitory effects of inflammasomes and their mutual regulation as potential mechanisms for achieving innate immune cell activation homeostasis (see **Figure 1**). A recent investigation suggests NLRC4 exerts a protective effect by regulating NLRP3-dependent cytokine production. NLRC4 inhibits prostaglandin E2 production, reducing IL-1β release from macrophages and dendritic cells in the lungs. This increases mortality in mice, indicating NLRC4 promotes disease by regulating inflammatory cytokine and cellular responses dependent on NLRP3 inflammasome activity (43). Competition for binding between NLRC3 and NLRP3 inflammasomes inhibits pro-inflammatory cytokine maturation and secretion, evidenced by decreased intracellular and released IL-1β. Additionally, NLRC3 overexpression decreased NLRP3-induced ASC speck formation in a dose and time dependent manner. Therefore, NLRC3 inhibits NLRP3 inflammasome complex formation by inhibiting NLRP3-ASC interaction or pro-IL-1β maturation (44). Upon NLRP3 knockout, AIM2 inflammasomes induce caspase-1-dependent pyroptosis, triggering a compensatory pathway for pyroptosis in NLRP3 macrophages. Propofol activates pro-caspase-1 via NLRP3 and AIM2 inflammasomes mediated by the ASC junction of inflammasomes (45). Studies also found AIM2 binds unprocessed caspase-1, inducing pore formation and K+ efflux. Further AIM2 amplifies infection signals to trigger NLRP3 inflammasome activation (46). Research found NLRP12 cooperates with NLRP3 and NLRC4 to initiate pyroptosis by cleaving GSDMD through caspase-1 *in vivo* and *in vitro*. Additionally, IL-1β amplifies pyroptosis by promoting CASP8-HIF-1α-induced activation of NLRP12/NLRP3/NLRC4, indicating a role in a key positive feedback loop accelerating neuroinflammation and pyroptosis (47). The crosstalk between these inflammasomes warrants further detailed research, including mechanisms of direct and indirect regulation of inflammasomes and restoration of homeostasis via inflammasome interactions.

## 3 Pyroptosis and IBD

IBD is a chronic inflammatory disease of the intestine, primarily encompassing ulcerative colitis (UC) and Crohn’s disease (CD). The etiology of IBD remains unclear, generally believed to result from a combination of multiple environmental factors and genetic inheritance, leading to excessive and abnormal immune responses in the intestine (48). It’s reported that pyroptosis levels in the colon tissue of NLRP3 deficient mice are downregulated significantly resisting IBD occurrence (49). Research found that the level of butyrate producing bacteria Roseburia integralis in UC patients significantly decreased, and the flagellar protein of Roseburia integralis could inhibit NLRP3 inflammasome activation and pyroptosis in macrophages, alleviating UC (50). Recently, studies found that scutellarin inhibits caspase-11 activation and pyroptosis in macrophages by regulating the PKA signaling pathway. Further research found that scutellarin dose-dependently inhibits intracellular LPS-induced caspase-11 activation and pyroptosis, inhibiting non-canonical NLRP3 inflammasome activation (51). Research found that NLRP3 gene knockout mice have an increased risk of acute and recurrent colitis, but no significant difference in disease progression exists between NLRC4 deficient mice and wild-type mice. Therefore, NLRP3 inflammasomes play a crucial role in preventing and treating colitis, potentially therapeutic targets for IBD. A recent study also found that salidroside protects experimental colitis by inhibiting macrophage pyroptosis, regulating gut microbiota diversity, and balancing Th17/Treg (52). In summary, intestinal inflammation is a complex problem. Exploring the synergistic effects of pyroptosis with gut microbiota or mucosal barrier represents a new research frontier in IBD.

## 4 Pyroptosis and CRC

CRC is a disease associated with genetic mutations, ethnicity, age, family history, and lifestyle. Due to the gradual deterioration of the aging population and environment, CRC has become the third most common malignant tumor worldwide (53). The expression of inflammasome signaling and pyroptosis related genes in humans is regulated by various factors, including genetic, mutational, and environmental factors, ultimately affecting gene expression and inflammasome activation. For example, single nucleotide polymorphisms in the NLRP3 gene are associated with poor survival in patients with invasive CRC (54). Similarly, several single nucleotides in the NLRP3 gene are associated with CD, the main risk factor for CRC development. In addition, the AIM2 gene has microsatellite instability sites, leading to gene mutations related to CRC and small intestine cancer (55). Research has found that silencing of GSDMC leads to a significant decrease in proliferation and tumorigenesis of CRC cell lines, while overexpression of GSDMC promotes proliferation and tumorigenesis of CRC cell lines (56). These results indicate that GSDMC, as an oncogene, may be a potential therapeutic target for CRC. Other studies have found that regardless of age or tumor stage, the combination of two CpGs can accurately distinguish CRC from normal tissue, suggesting that GSDME is a promising new diagnostic indicator for CRC (57). Additionally, research has found that lack of NLRP3 inflammasomes can also lead to impaired IL-18 signaling in NK cells and promote CRC cell growth and metastasis. Mice lacking NLRP3 or caspase-1 are highly sensitive to AOM/DSS induced inflammation and have significantly increased risk of colon tumors (58). Due to significantly decreased IL-18 levels in mice lacking NLRP3 inflammasomes. Defective inflammasome activation leads to loss of colonic epithelial integrity, infiltration of large amounts of white blood cells, and increased chemokines, resulting in higher mortality in mice. Moreover, mice lacking ASC and caspase-1 are also susceptible to DSS-induced colitis and colon associated CRC (59). This provides evidence for the protective effect of inflammasomes in the inflammatory model of CRC. Therefore, pyroptosis plays a crucial role in CRC.

## 5 New research frontiers of pyroptosis in intestinal inflammation and cancer

Recent studies have demonstrated the feasibility and clinical potential of pyroptosis as an immune mechanism for intestinal inflammation and cancer. Many researchers are attempting to combine pyroptosis with other mechanisms to regulate immunity, homeostasis, and gut microbiota for therapeutic purposes by regulating pyroptosis. In the process of intestinal inflammation and cancer development, pyroptosis is activated and involves many aspects, including gut microbiota, metabolism, intestinal mucosal barrier, apoptosis, antioxidant and autophagy. In this article, the focus is on the correlation between pyroptosis in the development of intestinal diseases and the gut microbiota or intestinal mucosal barrier.

### 5.1 Pyroptosis and gut microbiota

The gut microbiota is a dense microbial community continuously exposed to the gastrointestinal tract. The composition and role of the gut microbiota has been studied for decades, with a widespread belief it plays an important role in host homeostasis maintenance (60). The gut microbiota directly affects host nutrient absorption and the immune system, with interrelationships maintaining balanced stability in the intestine. Disruption of this balance can trigger, excessive or abnormal immune responses, leading to inflammatory diseases like IBD and CRC (61, 62).

Many studies report a significant decrease in gut microbiota biodiversity in gut tissue samples of IBD patients, resulting in a reduced total species community. Compared to healthy individuals, IBD patients exhibit significantly reduced gut microbiota biodiversity across different intestinal regions. These differences correlate significantly with disease activity (63). The causal relationship between changes in gut microbiota diversity and IBD pathogenesis remains elusive, with most studies focusing on microorganism-IBD interactions through immune responses. Inflammasome associated proteins are expressed in immune cells (e.g., macrophages, dendritic cells) and non-immune cells (e.g., intestinal epithelial cells, fibroblasts). Given their role as microbial sensors, inflammasome activation by gut microbiota may lead to physiological and pathological consequences for the host (23).

Reports indicate that NLRP1 exacerbates colitis through interactions with symbiotic microorganisms in animal models of IBD. Research found NLRP1 inflammasome can also regulate gut microbiota of co-litter control mice. Further, NLRP1 activated IL-18 contributes to DSS-induced colitis phenotype. IL-18 expression level in human intestinal biopsy samples negatively correlates with Clostridium population (25). NLRP1 inflammasome inhibits beneficial bacteria via IL-18, which may inhibit the growth of butyrate producing bacteria by inducing antimicrobial peptides. A decrease in the number of butyrate producing bacteria can damage intestinal barrier function (64).

Some results indicate mutual regulation between the NLRP3 inflammasome and gut microbiota. When NLRP3 inflammasome is activated by pathogens such as bacterial toxins, reactive oxygen species, ATP, etc. in the intestine, its downstream caspase-1 effector protein activates inflammatory factors such as IL-1β and IL-18, thereby triggering an intestinal inflammatory response. According to reports, MCC950 can increase the abundance of Firmicutes and reduce the abundance of Bacteroidetes, increasing the ratio of Firmicutes to Bacteroidetes, indicating that inhibition of NLRP3 inflammasomes can regulate gut microbiota composition. Further correlation analysis revealed that NLRP3 may play a functional role in regulating oxidative indicators by altering gut microbiota, especially Bacteroidetes, Lactobacillus reuteri and Lactobacillus genus (65). In addition, studies have found that Bacillus cereus regulates the TLR4-NF-κB-NLRP3 inflammasome signaling pathway in the intestinal mucosal barrier by regulating gut microbiota composition, thereby alleviating colitis (66).

According to reports, NLRC4 inflammasome activation is induced by translocation of a small amount of flagellin from Gram-negative pathogens into the host cytoplasm (67). Thus, in macrophages, NLRC4 can be activated by cytoplasmic flagellin derived from Salmonella typhimurium. In addition to Salmonella, NLRC4 can also recognize other Gram-negative bacteria, including Pseudomonas aeruginosa, Shigella flexneri and Legionella pneumophila (68). Furthermore, there was an increase in pyroptotic cells in mice colonized with Escherichia coli (E. coli), which directly and indirectly induced inflammatory macrophages via NLRC4, caspase8, and caspase1/11 complexes. This potentially promotes microbial translocation to distant tissues or organs, revealing new mechanisms of how colitis relates to the gut microbiota, leading to inflammatory macrophages in the gut, extraintestinal tissues and organs. Inflammatory macrophages are associated with various systemic diseases. Thus, researchers suggest chronic inflammatory disease occurrence and development may relate to inflammatory macrophages (69).

Like other inflammasomes, AIM2 inflammasomes also regulate gut microbiota (70). AIM2 inflammasomes can sense microbial DNA from gut bacteria, leading to IL-18 secretion in the intestine. Lack of AIM2 results in imbalanced gut ecology and increased susceptibility to DSS-induced colitis (40). Further research suggests AIM2 inflammasomes are part of host defense against excessive symbiotic E. coli growth in the gut. Notably, the colitis phenotype of AIM2 deficient mice is not entirely E. coli dependent, as despite high E. coli burden, AIM2 deficient mice still exhibit reduced colitis symptoms, indicating other unidentified bacteria migrating from wild-type to AIM2 deficient mice may protect the mouse colon (40).

### 5.2 Pyroptosis and intestinal mucosal barrier

The intestinal epithelial barrier (IEB) is one barrier between the environment and the internal environment. The IEB dynamically receives and responds to various stimuli, making it essential as the first line of intestinal defense. On one hand, it limits passage of harmful antigens and microorganisms. On the other, it ensures absorption of nutrients and water. Maintaining this delicate balance is strictly regulated, critical for body homeostasis. This balance is achieved through joint action of several parts, including mucus, a single-cell layer of epithelial cells, and an underlying layer of immune cells such as dendritic cells, macrophages, plasma cells, and lymphocytes (71). IEB dysfunction is related to pathogenesis of intestinal-related diseases such as IBD, irritable bowel syndrome, and CRC.

The mucous layer covers the intestinal lumen surface and serves as the first defense against physical, chemical, and biological damage. It protects intestinal epithelial cells from digestive enzymes, bacteria, and hazardous substances from external sources, including environmental pollutants and toxins (72, 73). Additionally, the mucus layer lubricates the food channel and removes bacteria and food debris from the intestine. It also provides a physical barrier and is crucial for maintaining intestinal homeostasis, as gases, small molecules, ions, and water can diffuse through the mucus layer to reach the epithelium (74). Reports show that the specific loss of GSDMD in intestinal epithelial cells results in decreased intestinal mucus secretion, loss of the mucus layer, and significant changes in the spatial distribution and composition of the intestinal microbiota (75). The colonic mucus layer not only harbors symbiotic bacteria, but also serves as an effective physical barrier against intestinal pathogens like E. coli. Mice with GSDMD deficiency in intestinal epithelial cells are more susceptible to intestinal pathogen infection. Further research found that GSDMD function in goblet cells limits pathogen colonization rate and deeper penetration into crypts by providing a physical barrier (75, 76).

Below the mucus layer lies a columnar monolayer of intestinal epithelial cells, providing another barrier between the external environment and the intestinal mucosa. IEB dysfunction allows excessive passage of food and microbiota antigens, triggering immune activation linked to intestinal and systemic diseases. IEB function depends on a series of intercellular connections composing the apical junction complex (AJC), including tight junctions (TJs), desmosomes and adhesive junctions (AJs). Under physiological conditions, only solutes like water and electrolytes pass paracellularly through the epithelium. TJs are the main regulators of paracellular permeability, forming a protein network atop epithelial lateral membranes, including claudins, tight junction related marvel proteins (TAMPs) like junctional adhesion molecule A (JAM-A), occludin, and intracellular scaffold proteins such as tricellulin and zonula occlusion (ZO) (77, 78). According to reports, GSDMD mediated pyroptosis is activated in both lung and intestinal mucosal tissues during LPS-induced acute lung injury. GSDMD knockout mice improved LPS-induced acute lung injury related intestinal mucosal barrier damage, confirming GSDMD-mediated pyroptosis’s key role in acute lung injury and intestinal mucosal dysfunction. Further GSDMD deficiency mitigated intestinal mucosal dysfunction induced by acute lung injury (79). GSDMD is thus a potential targeted treatment for intestinal mucosal injury. Additionally, calcitriol effectively alleviated hyperosmotic stress-induced corneal epithelial cell damage by inhibiting the NLRP3-ASC-Caspase-1-GSDMD pyroptosis pathway (80). It is thus urgent to confirm GSDMD’s role in new intestinal mucosal damage and explore treatments for intestinal inflammation and cancer.

## 6 Conclusions

This article provides a comprehensive review of the mechanisms and functions of pyroptosis and inflammasomes in intestinal inflammation and cancer. However, our current understanding of pyroptosis in these conditions is limited, with many unanswered questions remaining. One area of inquiry is how different inflammasomes collaborate in the context of intestinal inflammation and cancer. The causal relationship between changes in gut microbiota diversity and the pathogenesis of IBD requires further exploration. Another intriguing question is whether GSDMD could serve as a potential target for repairing intestinal mucosal damage in these conditions. Furthermore, the specific mechanisms of action of various components of pyroptosis in intestinal inflammation and cancer remain unclear. Addressing these knowledge gaps and elucidating potential molecular mechanisms will pave the way for developing new therapies and diagnostic approaches. Therefore, understanding the role of inflammasomes and pyroptosis in intestinal inflammation and cancer is paramount. In conclusion, this review highlights growing evidence supporting the involvement of pyroptosis in intestinal inflammation and cancer.

## Author contributions

LC: Conceptualization, Writing – original draft, Writing – review & editing. WZ: Conceptualization, Writing – review & editing. YF: Conceptualization, Writing – review & editing. PG: Formal analysis, Writing – review & editing. JM: Formal analysis, Writing – review & editing.
